# Synergy between a shallow root system with a *DRO1* homologue and localized P application improves P uptake of lowland rice

**DOI:** 10.1038/s41598-021-89129-z

**Published:** 2021-05-04

**Authors:** Aung Zaw Oo, Yasuhiro Tsujimoto, Mana Mukai, Tomohiro Nishigaki, Toshiyuki Takai, Yusaku Uga

**Affiliations:** 1 Japan International Research Center for Agricultural Sciences, 1-1 Ohwashi, Tsukuba, Ibaraki 3058686 Japan; 2 Institute of Crop Science, National Agriculture and Food Research Organization (NARO), 2-1-2, Kan-nondai, Tsukuba, Ibaraki 3058518 Japan

**Keywords:** Plant breeding, Plant physiology, Abiotic

## Abstract

Improved phosphorus (P) use efficiency for crop production is needed, given the depletion of phosphorus ore deposits, and increasing ecological concerns about its excessive use. Root system architecture (RSA) is important in efficiently capturing immobile P in soils, while agronomically, localized P application near the roots is a potential approach to address this issue. However, the interaction between genetic traits of RSA and localized P application has been little understood. Near-isogenic lines (NILs) and their parent of rice (*qsor1*-NIL, *Dro1*-NIL, and IR64, with shallow, deep, and intermediate root growth angles (RGA), respectively) were grown in flooded pots after placing P near the roots at transplanting (P-dipping). The experiment identified that the P-dipping created an available P hotspot at the plant base of the soil surface layer where the *qsor1*-NIL had the greatest root biomass and root surface area despite no genotyipic differences in total values, whereby the *qsor1*-NIL had significantly greater biomass and P uptake than the other genotypes in the P-dipping. The superior surface root development of *qsor1*-NIL could have facilitated P uptakes from the P hotspot, implying that P-use efficiency in crop production can be further increased by combining genetic traits of RSA and localized P application.

## Introduction

Phosphorous deficiency restricts crop growth, particularly in the tropics, due to the inherently low P content of soils and the high P-fixing capacity of other minerals such as active Al- and Fe- oxides^1^. Large amounts of mineral P fertilizer have been continuously applied to overcome low P-use efficiency and achieve high grain yields. Given the finite nature of the P fertilizer resource and increasing ecological concerns about the excess use of P in agricultural systems^2–4^, it is vital to investigate sustainable crop production strategies that facilitate the efficient utilization of applied and available P in soils. Such strategies are also critical for the food security of resource-poor farmers with low fertilizer inputs in developing countries^5^.

Roots play a pivotal role in exploring immobile P in the soil. An increased root surface area with minimal carbon costs is one strategy, through the formation of finer roots, aerenchyma, and root hairs^6–8^. Changes in root system architecture (RSA) such as the development of surface roots is another root function to adapt to P deficiency, that is called ‘topsoil foraging’, because P is most available in surface soil layers^9^. This topsoil foraging can be enhanced by a shallower growth angle of axial roots^9^, adventitious root abundance^10^, and many/short lateral root branching^11^. Field-based studies have demonstrated the yield advantages of genotypes with these architectural traits for several crops under P-deficiency^8^. Therefore, identification of key root traits and their genetic mechanisms and conferring genes or quantitative trait loci (QTL) should offer avenues for improving P acquisition efficiency in crop breeding^12^.

The agronomic approach for improving P-use efficiency includes localized fertilization, which refers to the placement of small amounts of fertilizers nearby the root zone. Several field experiments have demonstrated the positive impacts of localized P fertilization on grain yields and/or fertilizer use efficiencies for crop production (e.g., Vandamme et al.^13^). Our recent study identified that applied P-use efficiency can be substantially improved by dipping seedling roots in P-enriched slurry at transplanting (P-dipping) in severely P-deficient rice fields in Madagascar^14^. The P-dipping transfers P with the slurry attached to seedling roots, creating a soluble P hotspot nearby the transplanted roots and facilitating plant P uptake, even under the high P-fixing soils of the tropics^15^. The use of P-dipping is currently being tested by hundreds of smallholder farmers in Madagascar.

Despite a range of studies in both genetic and agronomic approaches, none have examined how the combination of RSA traits and localized fertilization would affect plant P-use and acquisition efficiencies. In the present study, we aimed to identify the combination effect by using near-isogenic lines (NILs) of *DRO1*, and its homologue (*qSOR1*), the major QTLs of rice controlling root growth angle (RGA). The parent variety, ‘IR64’, is a high-yielding, modern variety with a relatively shallow RGA with the combination of the nonfunctional allele of *DRO1*, and the functional allele of *qSOR1*. The *Dro1*-NIL, developed by Uga et al.^16^, has a relatively deep RGA with the combination of functional alleles of both *DRO1* and *qSOR1*. The *qsor1*-NIL, developed by Kitomi et al.^17^, has a shallower RGA than IR64, with the combination of nonfunctional alleles of both *DRO1* and *qSOR1*. We hypothesize that P-dipping, creating a P hotspot at the soil surface, will have a positive interaction with the shallow root system in rice. By understanding the interaction, further research can be expected to improve applied P-use efficiencies by designing RSA traits for localized fertilizer application techniques.

## Results

### Shoot growth and P uptake

**Figure 1:**
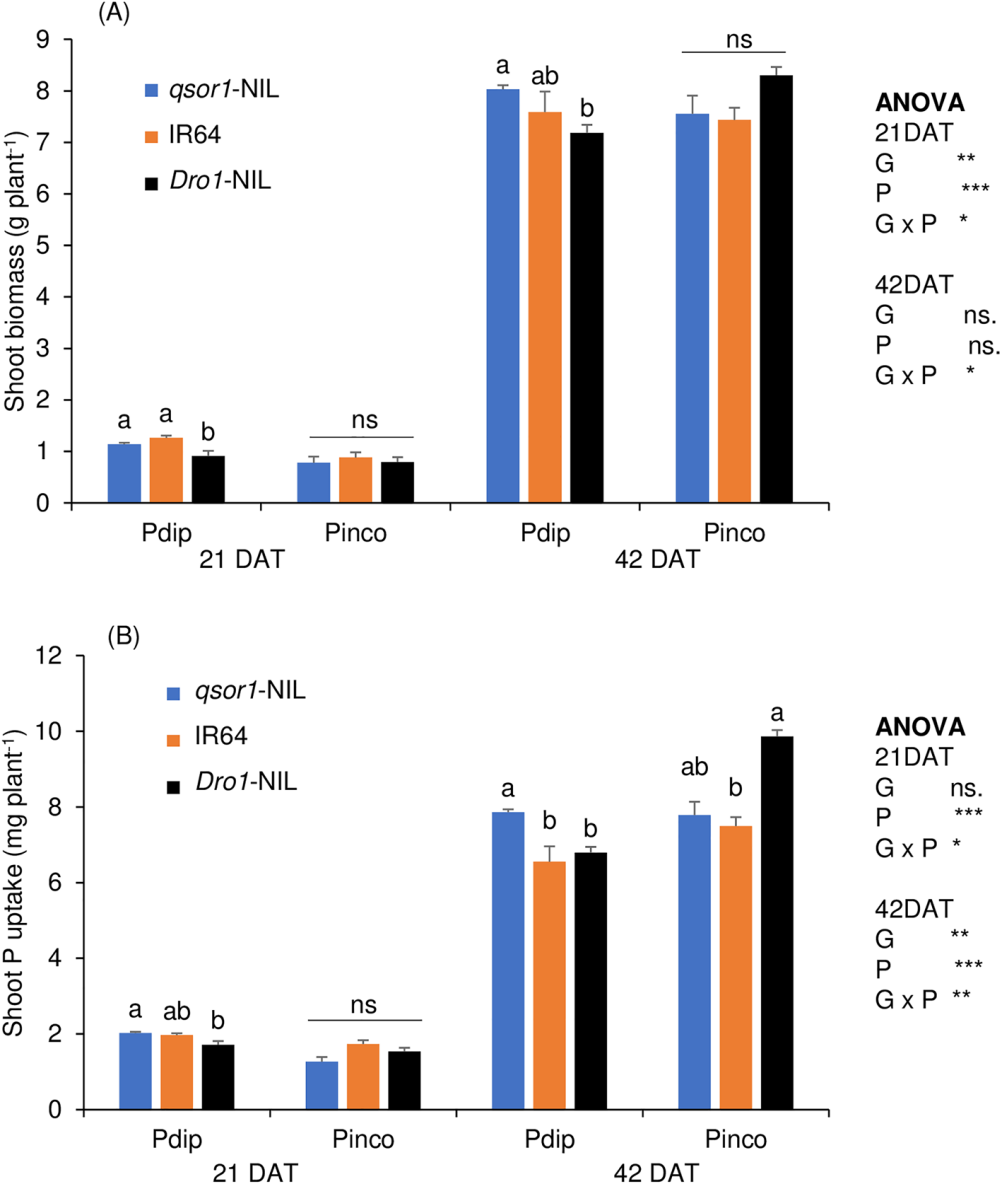
Shoot biomass (**A**) and shoot P uptake (**B**) of rice genotypes as affected by different P application methods (P incorporation (P_inco_) of 500 mg P_2_O_5_ box^−1^ vs. P-dipping (P_dip_) of 90 mg P_2_O_5_ box^−1^) at 21 days after transplanting (DAT), and 42 DAT. Different letters and ns within each treatment indicate significant and non-significant differences, respectively, among genotypes at 5% using Tukey's HSD test. Error bars represent the standard error of replications. The *, **, and *** indicate that the individual effects of and interaction between genotype (G) and P application method (P) are significant at P < 0.05, P < 0.01, and P < 0.001, respectively.

Localized P application via P-dipping (P_dip_) achieved equivalent biomass and P uptakes at one fifth of the application rate of uniform P incorporation (P_inco_) (Fig. 1). The ANOVA detected consistent and significant interactions between genotype and P treatment for shoot biomass and P uptakes at both 21 days after transplanting (DAT) and 42 DAT. In the P_dip_ treatment, *qsor1*-NIL consistently had greater shoot biomass and P uptake than *Dro1*-NIL. In contrast, in P_inco_, *Dro1*-NIL tended to have greater shoot biomass and significantly greater P uptakes than the other genotypes at 42 DAT. Applied P-use efficiency (calculated as the ratio of shoot P uptake at 42 DAT to the amount of P applied) increased from 3.4 to 16.2% for IR64 by changing the P application methods from P incorporation to P-dipping and further increased to 20.0% by using *qsor1*-NIL (data not shown).

### Genetic root traits

**Figure 2:**
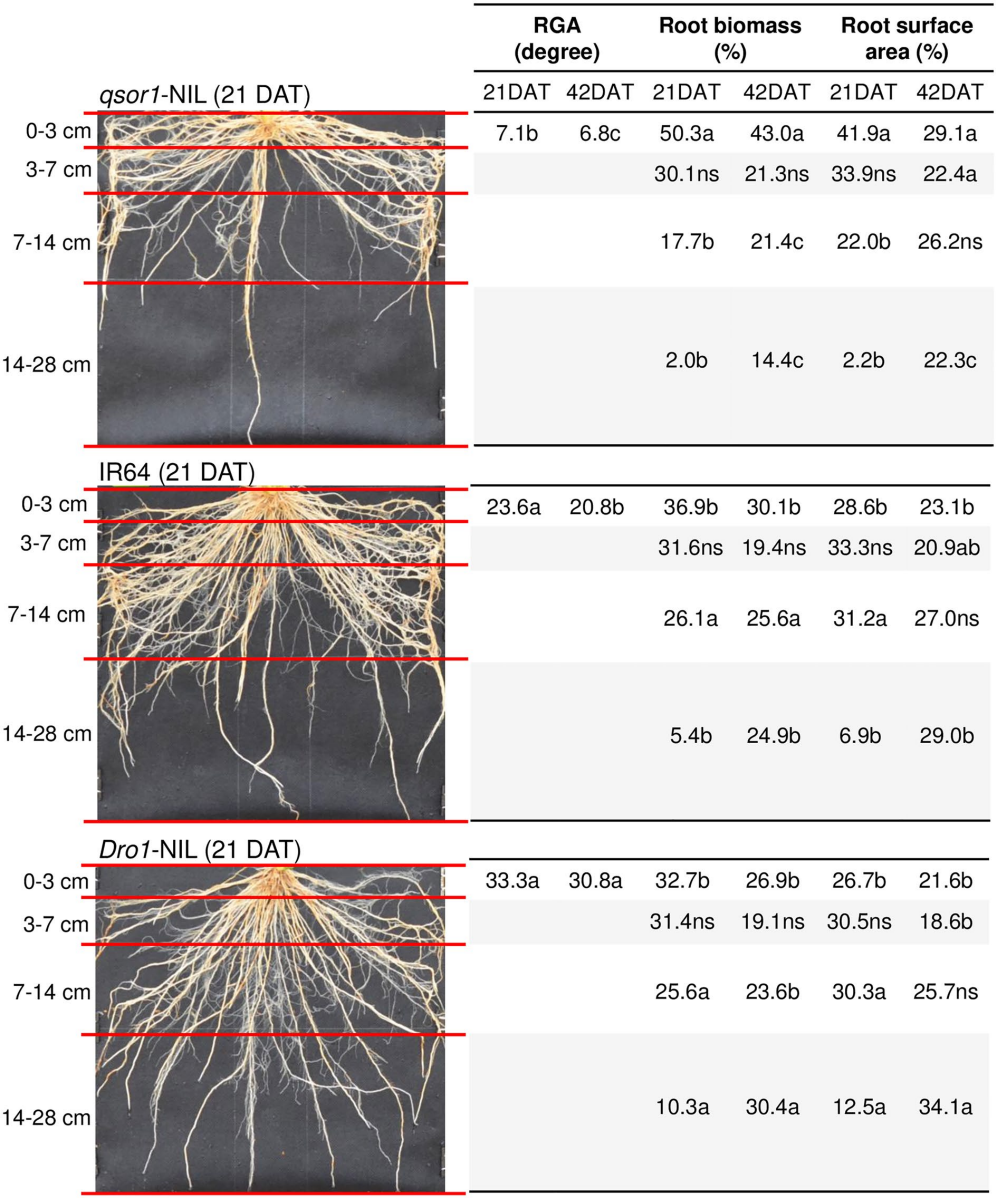
Root growth angle (RGA) and proportions of root biomass and root surface area in different soil layers of *qsor1*-NIL, IR64, and *Dro1*-NIL at 21 days after transplanting (DAT), and 42 DAT under the P-dipping (P_dip_) treatment. Different letters in the same soil layer indicate significant differences among genotypes at 5% of Tukey's HSD test. *ns* not significant at 5% level.

**Figure 3:**
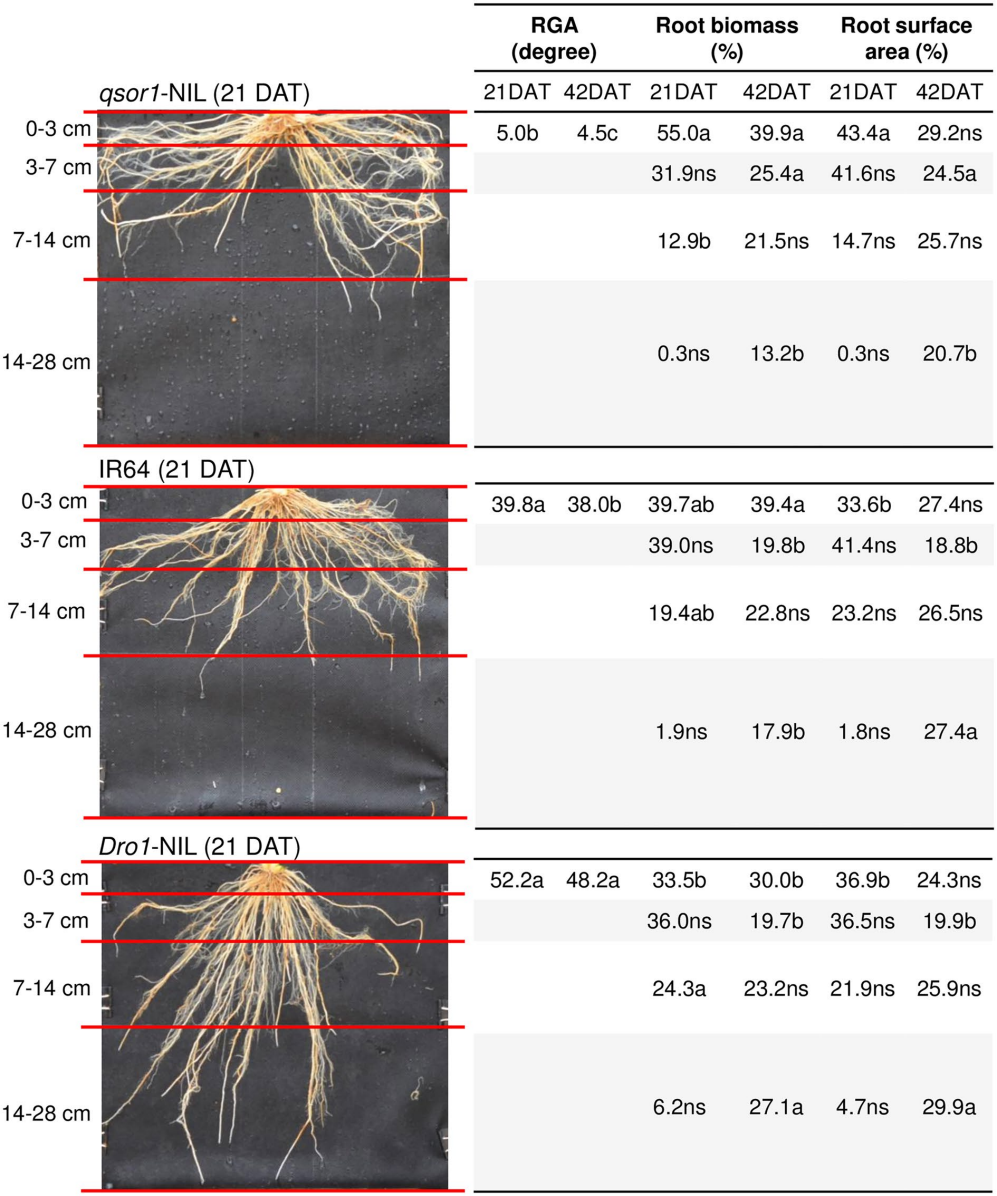
Root growth angle (RGA) and proportions of root biomass and root surface area in different soil layers of *qsor1*-NIL, IR64, and *Dro1*-NIL at 21 days after transplanting (DAT), and 42 DAT under the P incorporation (P_inco_) treatment. Different letters in the same soil layer indicate significant differences among genotypes at 5% of Tukey's HSD test. *ns* not significant at 5% level.

The RSA traits among genotypes were consistent under P_dip_: the RGA was the shallowest in the order of *qsor1*-NIL > IR64 > *Dro1*-NIL at both 21 DAT and 42 DAT (Fig. 2). As a result of the RGA differences, *qsor1*-NIL developed a large proportion of root biomass and root surface area in the 0–3 cm layer and little in the 14–28 cm layer. In contrast, *Dro1*-NIL distributed a relatively large proportion of root mass in the 14–28 cm layer. For instance, at 21 DAT, *qsor1*-NIL developed 50.3% of the root mass in the 0–3 cm layer and only 2.0% in the 14–28 cm layer while these proportions were 32.7% and 10.3%, respectively, for *Dro1*-NIL. The root distribution pattern of IR64 was intermediate between *qsor1*-NIL and *Dro1*-NIL. The trend in RSA among genotypes was the same in P_inco_ while IR64 and *Dro1*-NIL tended to have deeper RGAs than those in P_dip_ (Fig. 3). The RGAs of *qsor1*-NIL, IR64, and *Dro1*-NIL at 21 DAT were 7.1°, 23.6°, and 33.3° in P_dip_ and 5.0°, 39.8°, and 52.2° in P_inco_, respectively.

**Figure 4:**
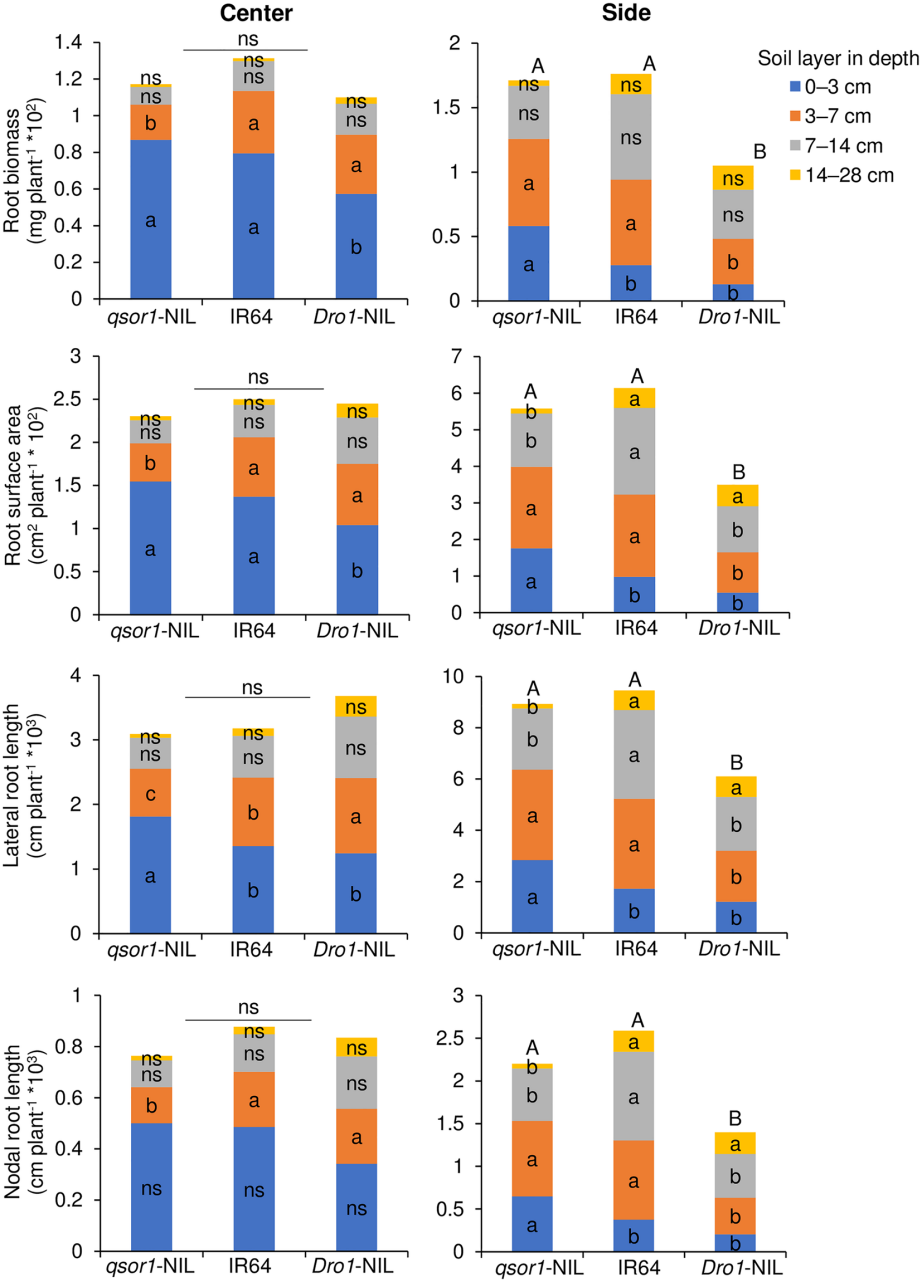
Horizontal and vertical root distribution patterns of three rice genotypes at 21 days after transplanting under the P-dipping (P_dip_) treatment. Root parameters were shown in the center and side positions of four different soil layers (0–3 cm, 3–7 cm, 7–14 cm, and 14–28 cm). The center position indicates 3 cm from the plant base in both horizontal directions. The side position is apart from the center position. Different small letters and capital letters indicate significant differences among genotypes in these parameters within each soil layer and in total of all layers, respectively, at 5% of Turkey's HSD test. *ns* not significant at 5% level.

**Figure 5:**
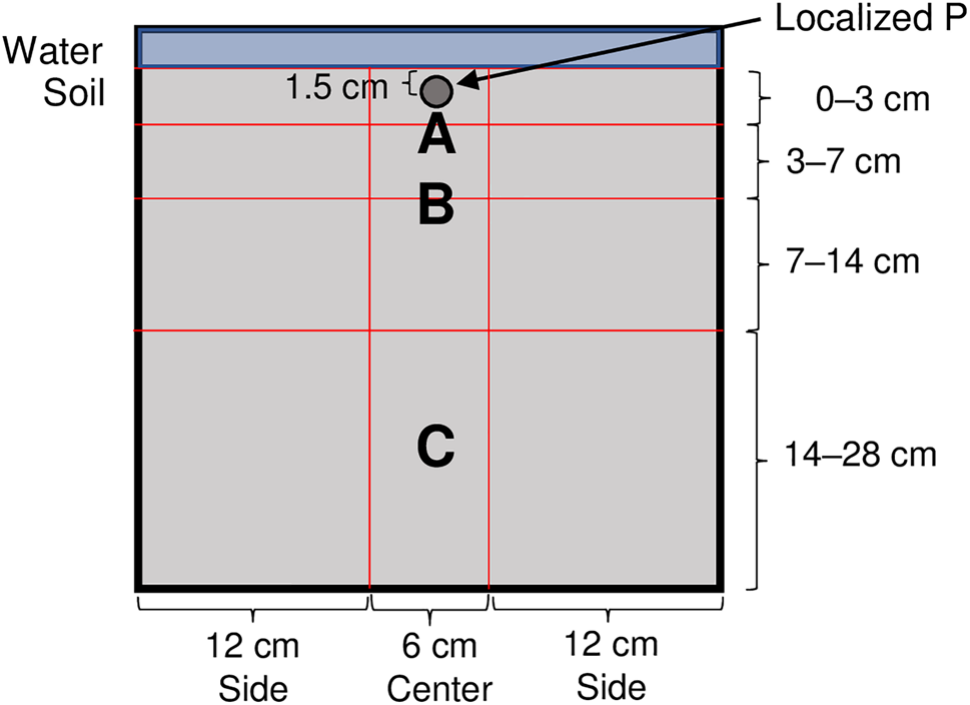
Schematic representation of the root box (30 cm × 30 cm × 3 cm) with the sampling points of soil water solution. Ceramic Rhizon samplers were installed in the middle of the box at (**A**) 3 cm, (**B**) 7 cm, and (**C**) 21 cm depths. The observation at 7 cm (**B**), and 21 cm (**C**) depth was conducted in both treatments while the observation at 3 cm depth (**A**) was only conducted in the P-dipping (P_dip_) treatment.

By reflecting on the differences in RGA, *qsor1*-NIL had a greater root biomass, greater root surface area, and longer lateral root length than *Dro1*-NIL at both the center position (0 to 3 cm from the plant base on both sides), and side position (3 cm from the plant base to the edges of the box) in the 0–3 cm soil depth layer at 21 DAT (Figs. 4; 5). On the other hand, *Dro1*-NIL had greater root surface area and longer lateral and nodal root length than *qsor1*-NIL at the side position in the 14–28 cm layer despite its significantly lower values in total for these parameters at 21 DAT (the mean values were also higher in the center position, but the differences were not statistically significant) (Fig. 4). IR64 was intermediate for these parameters in both the 0–3 cm and 14–28 cm layers. At 42 DAT, genotypic differences in root distribution patterns vertically and horizontally became less significant as root development and root growth angle were increasingly constrained by the size of the root box (Fig. S1). Yet, *qsor1*-NIL had consistently greater root mass and greater root surface area than *Dro1*-NIL at the side position in the 0–3 cm layer and vice versa in both center and side positions in the 14–28 cm layer.

### Spatio-temporal dynamics in soluble P concentrations

**Figure 6:**
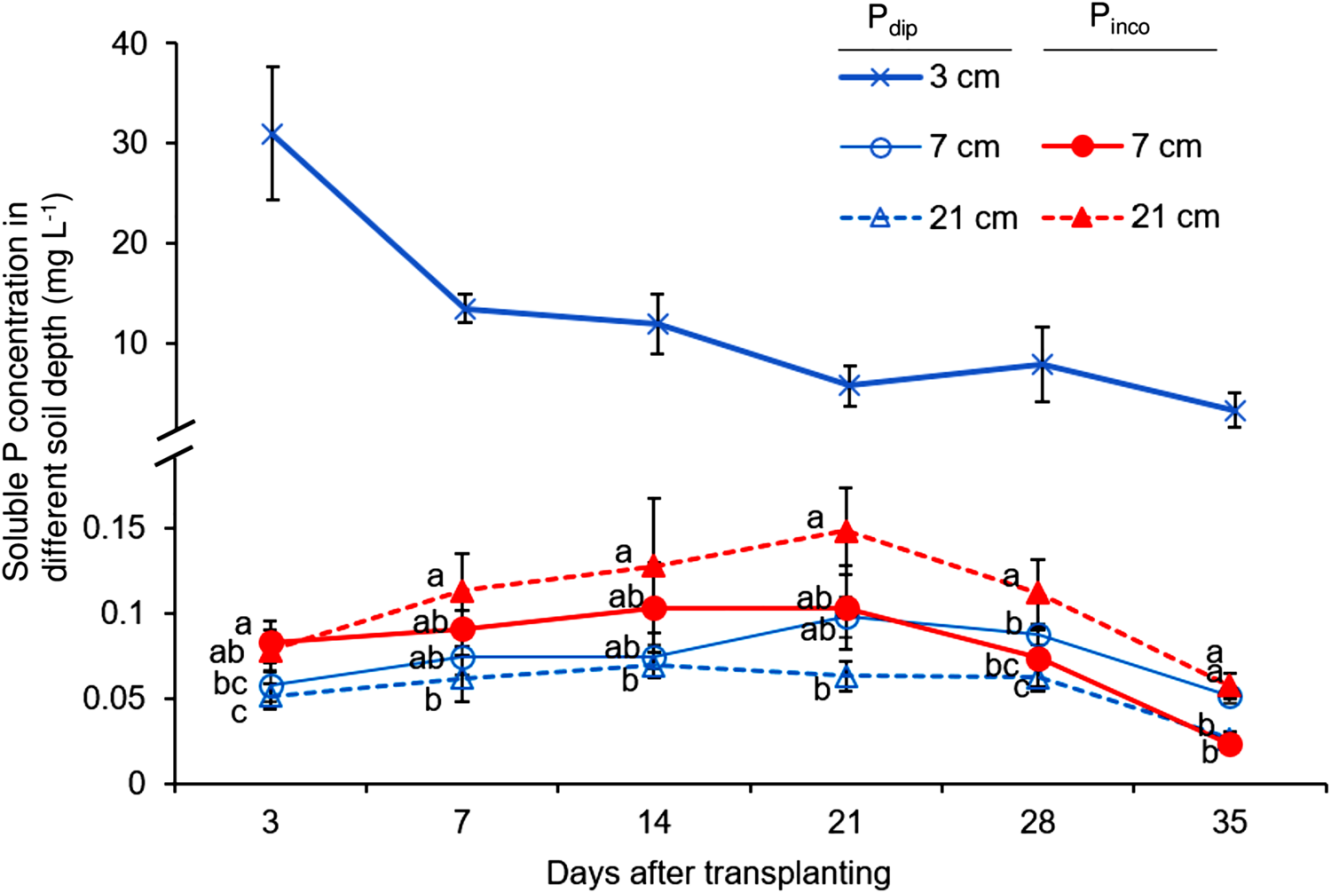
Spatio-temporal variations in soluble P concentration as affected by different P application methods. The cross symbols indicate the value at the 3 cm depth of the P-dipping (P_dip_) treatment. The open and closed circles indicate the value at the 7 cm depth of the P_dip_ treatment, and P incoropration (P_inco_) treatment, respectively. The open and closed triangles indicate the value at the 21 cm depth of the P_dip_ treatment and P_inco_ treatment, respectively. Data values are an average of three rice genotypes because no significant genotype difference in soluble P concentration was observed at each sampling time. Error bars indicate standard error of replications. Different letters indicate significant differences at 5% using Tukey's HSD test among different soil depths (7 cm and 21 cm) by P application methods. The observation at 3 cm depth was only conducted in the P_dip_ treatment.

Soluble P concentrations in soils were averaged across genotypes because there were no significant genotype differences in any sampling times or sampling layers. The P_dip_, in which high P solution was applied by spot at a depth of 1.5 cm from the soil surface, had a substantially large soluble P concentration at a depth of 3 cm (Fig. 6). The maximum P concentration at a depth of 3 cm for P_dip_ was > 100 times greater than the other depths for both P treatments throughout the growing period. In P_dip_, soluble P concentrations were greater at a depth of 7 cm than at 21 cm in the latter growth stages, but apparently the vertical P diffusion from the 3 cm hotspot was relatively small. In contrast, the soluble P concentrations were significantly higher at a depth of 21 cm than at 7 cm in P_inco_ after 28 DAT.

## Discussion

The results support the hypothesis that the shallow root system of *qsor1*-NIL has a positive interaction with localized P application via P-dipping and that the combination additively improves applied P-use efficiency for initial rice growth. The other genotypes also reduced the RGA by 16–19° in response to the P hotspot (Figs. 2, 3), yet the synergy with P-dipping was greater in *qsor1*-NIL. This implies that breeding efforts to design the RGA in localized P spots can be more beneficial than relying on the intrinsic root plasticity of each genotype. It is also considered that root phenotypic adaptations to growing environments (as found in RGA changes of the other genotypes) may have a certain metabolic cost^18^.

Superior P uptake of *qsor1*-NIL with P-dipping is attributable to the greater root biomass, greater root surface area, and longer lateral root length, especially at the center position in the 0–3 cm soil layer where high soluble P is available throughout the growing period. This is most likely the same mechanism as topsoil foraging, prioritizing the root development in the P-rich domains to efficiently capture immobile P in soils. Spatio-temporal P variations in the P-dipping indicate that applied P mobility is highly restricted despite a general understanding that P becomes less immobile under flooded conditions^19^, emphasizing the importance of RSA for the localized P acquisition, even under flooded soil culture. The relative immobility of applied P even in irrigated lowlands was also reported by Akahane et al.^20^, in which they detected high amounts of P retained up to harvest within a small area of 2–3 cm (vertical) by 4–5 cm (horizontal) centered on the application spot. This area of P distribution surrounding the application spot under the flooded soil culture corresponds to the center position of the 0–3 cm soil layer surrounding the P-applied spot of the P_dip_ treatment in the present study.

The effect of topsoil foraging itself has been reported in several upland crops^11,21–23^, but not in rice. Previous studies detected no significant effects of root distribution patterns or RGA for rice P acquisition under P deficiency (e.g., Mori et al.^24^), which may be due to the materials differing not only in root system architecture but in other traits or in more complex screening environments. The present study had an advantage in using NILs that differed in RGAs (but were otherwise equivalent phenotypes^17^), under non-water-stressed, and greatly uneven P availability because of P-dipping.

In addition, the present study detected a positive effect of *Dro1*-NIL on P uptake under uniform, P-sufficient conditions. The reason for this positive interaction should be further explored but can be related to consistent P acquisition from the P-rich subsoil layers after the depletion of available P in topsoil layers (Fig. 6). Another potential reason is the more efficient acquisition of other nutrients, such as N, which are vertically more mobile than P. Deep rooting has been reported as a positive trait for N acquisition in upland crops^8^ and also in rice in flooded paddy fields in the latter growth stages^25^. In the common bean, Rangarajan et al. postulated that the greater vertical range of roots with deeper RGA, and a greater number of basal root whorls is advantageous for biomass production when both N and P are deficient^26^. Likewise, dispersed root distribution of *Dro1*-NIL might have benefited from the relatively uniform nutrient conditions of the P_inco_ treatment. *Dro1*-NIL had significantly smaller coefficients of variation across soil layers in root biomass than *qSOR1* (58% vs. 98% at 21 DAT, and 23% vs. 47% at 42 DAT, respectively), indicating more uniform and dispersed root development.

It should be noted that crop production environments are complex with multiple abiotic stresses, particularly on smallholder farms in developing countries where stress-resilient and nutrient-efficient technologies are most needed. In this respect, field-based experiments to maturity are further required to confirm the effect of the combination of genetic RSA traits and P fertilizer management practices. The combination of shallow roots and localized P application can never be a silver bullet. A careful selection of field environments where P deficiency is the primary limiting factor is needed to effectively apply this combination, ideally together with the development of bimodal root phenotypes (shallow and deep), or high RSA plasticity^27^ against complex growing environments. In rice, *qSOR1* and *DRO1* can be promising genetic resources for the development of such bimodal root phenotypes, without increasing costs of root elongation but by controlling the root growth angles^28,29^. A combination of shallow root system and RSA plasticity may be another possible trait ensuring both efficient P uptake from localized spots, and flexible responses to unpredictable changes in growing environments.

## Conclusion

The study provides significant evidence that a shallow root system has a positive interaction with localized P application nearby the root at transplanting by using NILs differing in their RGA. The combination substantially improves applied-P use efficiency for initial rice growth. This finding should encourage relevant research focusing not only on physiological root traits or agronomic management approaches, but on their combination to address to the global issue of increasing crop production while minimizing the environmental impacts.

## Materials and methods

### Experimental design and treatments

The experiment was conducted in a greenhouse with an automatic ventilation system at the Japan International Research Center for Agricultural Science (JIRCAS), Tsukuba, Japan. The average daytime and nighttime temperatures during the experiment ranged from 26.2 to 35.8 °C and 24.7 to 28.7 °C, respectively (Thermo Recorder TR-50U2, T&D Corporation, Japan).

The soil for the experiment was collected from a subsoil layer (40–50 cm in depth) at the JIRCAS Tropical Agricultural Research Front, Okinawa, Japan. Physicochemical properties of experimental soil are summarized in Table 1. The soil was silty clay with a pH (H_2_O) of 4.86 and low available P content, and high P retention capacity with abundant active Al and Fe oxides. The soil was air-dried and passed through an 8 mm sieve prior to the experiment.

**Table 1:** Physicochemical properties of soil.

Parameters	Red-yellow soil
pH (H_2_O)	4.86
EC (mSm^−1^)	4.63
Total N (g kg^−1^)^a^	0.91
Total C (g kg^−1^)^a^	4.82
P retention (%)^b^	57.5
Available P (Bray II) (mg kg^−1^)^c^	17.5
P_oxalate_ (mg kg^−1^)^d^	207.5
Al_oxalate_ (g kg^−1^)^d^	2.70
Fe_oxalate_ (g kg^−1^)^d^	2.58
Sand (%)^e^	44.3
Silt (%)^e^	12.0
Clay (%)^e^	43.7
Texture	Silty clay

^a^NC analyzer, Sumigraph NC-220F (SCAS, Japan); ^b^The proportion of absorbed P after shaking 5 g of soil with 25 ml of 1000 ppm P solution for 24 h; ^c^UV spectrophotometer (UV-1800, Shimadzu); ^d^Inductively coupled plasma mass spectrometer (ICPE-9000, Shimazu, Japan) after oxalate extraction. ^e^Sieving and pipetting method.

Two different P treatments, sufficient P incorporation (P_inco_) and localized P application via P-dipping (P_dip_), were factorially combined with three rice genotypes in a randomized complete block design with seven replications. For both treatments, NH_4_NO_3_ and K_2_SO_4_ were mixed with soils and puddled in a bucket at a rate of 220 mg N box^−1^, and 220 mg K_2_O box^−1^ to develop uniform and N- and K-sufficient conditions. For the P_inco_, triple super phosphate (TSP) was added at puddling. The mixed soils were filled into a root box at a rate of 500 mg P_2_O_5_ box^-1^ to develop a uniform and P-sufficient condition. The P application rate of the P_inco_ treatment was determined based on Oo et al.^15^ to expect similar levels of plant P uptakes and biomass production with the P_dip_ treatment for the comparison of root development. The root box was made of transparent acrylic sheets with a size of 30 cm height × 30 cm length × 3 cm width. The soil was added to the box to a depth of 28 cm.

For the P_dip_ treatment, a P solution was placed in a spot nearby the transplanted root zone to apply the exact amount of P in all boxes. We estimated the amount of P-enriched slurry transferred or attached to seedling roots at transplanting as 90 mg P_2_O_5_ box^−1^ based on our previous study^30^. After the N- and K- added soil was filled in the root box, 90 mg P_2_O_5_ as TSP dissolved in 20 ml water was injected into the soil at a depth of 1.5 cm in the center of the root box (Fig. 5). On the same day of these P treatments, one 10-day old seedling was transplanted in the middle of each root box and grown under continuously flooded conditions.

### Measurement

Soil solution samplers (DIK-8393, Daiki Rika Kogyo Co. Ltd., Japan) were installed in one side of the acrylic board in the middle of the 3 cm, 7 cm, and 21 cm depths for the P_dip_ treatment and at 7 cm and 21 cm for the P_inco_ treatment for four out of the seven replicates (Fig. 5). Based on our previous observation^15^, we assumed that the spatial variation in soluble P concentration were relatively small in the box because we thoroughly mixed P with soil at the time of puddling and thus omitted the measurement at 3 cm in the P_inco_ treatment.

Soil water samples were collected at 3, 7, 14, 21, 28, and 35 DAT. The samples were analyzed for soluble P concentration as an index of P available to plants using a microplate reader spectrophotometer at an absorbance of 630 nm by following the Malachite Green method^31^.

Three and four replicates were harvested at 21 DAT and 42 DAT, respectively. At each harvest time, shoots were cut at ground level and oven-dried at 70 °C for > 48 h to determine shoot biomass. Shoot P concentration was measured with the molybdate blue method^32^ after dry-ashing at 550 °C for 2 h and digestion with 0.5 M HCl. Shoot P uptake was calculated by multiplying the P concentration and shoot biomass.

After shoots were removed, root samples were collected using pin-board method as per Kano-Nakata et al^33^. In brief, roots were pinned with a 5 mm mesh net and pinboard after which soils were washed off and digital images were taken. The RGA was determined from the digital image taken by a commercial camera (D7000, Nikon Corp., Japan) as the angle from the soil surface to the shallowest nodal root using ImageJ software (Version 1.52a, NIH, USA). The root system was then divided into 12 compartments or into the center (0 to 3 cm from the plant base) and both sides of the 0–3 cm, 3–7 cm, 7–14 cm, and 14–28 cm soil layers to assess spatial root distributions (Fig. 5). The values of both sides (left and right) were summed as there must be no physiological meaning in the difference between these two. Root length and surface area of each compartment were measured using Epson Pro-selection X980 Scanner and WinRhizo Pro software (Regent Instruments, Quebec, Canada). Roots were classified as lateral roots (< 0.2 mm) as per Sandhu et al.^27^ and nodal roots (0.2 to 2 mm) as per Kano-Nakata et al.^34^. Roots of > 2 mm were excluded from the analysis, as they were too large for a single root diameter and most likely occurred as a result of a measurement error. After the morphological analysis, root biomass of each compartment was determined by oven-drying at 70 °C for > 48 h.

### Statistical analysis

JMP software (v14.0.0, SAS Institute Inc., Japan) was used to perform the statistical analyses. The treatment means were compared at 5% level of probability using Tukey’s honestly significant difference (HSD) test after the single and/or interaction effects of genotypes and P treatment were confirmed by a generalized linear model.

### The use of plant materials

Near-isogenic lines (NILs) and their parent of rice (*qsor1*-NIL, *Dro1*-NIL, and IR64) that we used in the present experiment was transferred from National Agricultural Research Organization (NARO) to Japan International Research Center for Agricultural Sciences (JIRCAS) by the Joint Research Contract, and the experiment was conducted by compiling with the guideline and regulation of this contract.

## Supplementary Information

Supplementary Information

## References

[1] Walker TW, Syers JK (1976). The fate of phosphorus during pedogenesis. Geoderma.

[2] Vance CP, Uhde-Stone C, Allan DL (2003). Phosphorus acquisition and use: Critical adaptations by plants for securing a nonrenewable resource. New Phytol..

[3] Carpenter SR, Bennett EM (2011). Reconsideration of the planetary boundary for phosphorus. Environ. Res. Lett..

[4] Nedelciu CE, Ragnarsdottir KV, Schlyter P, Stjernquist I (2020). Global phosphorus supply chain dynamics: Assessing regional impact to 2050. Glob. Food Sec..

[5] Tsujimoto Y, Rakotoson T, Tanaka A, Saito K (2019). Challenges and opportunities for improving N use efficiency for rice production in sub-Saharan Africa. Plant Prod. Sci..

[6] Lynch JP, Ho MD (2005). Rhizoeconomics: Carbon costs of phosphorus acquisition. Plant Soil..

[7] Nestler J, Keyes SD, Wissuwa M (2016). Root hair formation in rice (*Oryza sativa* L.) differs between root types and is altered in artificial growth conditions. J. Exp. Bot..

[8] Lynch JP (2019). Root phenotypes for improved nutrient capture: An underexploited opportunity for global agriculture. New Phytol..

[9] Lynch JP, Brown KM (2001). Topsoil foraging—an architectural adaptation of plants to low phosphorus availability. Plant Soil..

[10] Miller CR, Ochoa I, Nielsen KL, Beck D, Lynch JP (2003). Genetic variation for adventitious rooting in response to low phosphorus availability: Potential utility for phosphorus acquisition from stratified soils. Funct. Plant Biol..

[11] Jia X, Liu P, Lynch JP (2018). Greater lateral root branching density in maize improves phosphorus acquisition from low phosphorus soil. J. Exp. Bot..

[12] Burridge JD (2019). A case study on the efficacy of root phenotypic selection for edaphic stress tolerance in low-input agriculture: Common bean breeding in Mozambique. Field Crops Res..

[13] Vandamme E (2018). Phosphorus micro-dosing as an entry point to sustainable intensification of rice systems in sub-Saharan Africa. Field Crop Res..

[14] Rakotoarisoa NM, Tsujimoto Y, Oo AZ (2020). Dipping rice roots in P-enriched slurry at transplanting significantly affects grain yield and phenology development in severely P-deficient fields in Madagascar’s central highlands. Field Crop Res..

[15] Oo AZ, Tsujimoto Y, Rakotoarisoa NM, Kawamura K, Nishigaki T (2020). P-dipping of rice seedlings increases applied P use efficiency in high P-fixing soils. Sci. Rep..

[16] Uga Y (2013). Control of RGA by DEEPER ROOTING 1 increases rice yield under drought conditions. Nat. Genet..

[17] Kitomi Y (2020). Root angle modifications by the DRO1 homolog improve rice yields in saline paddy fields. PNAS.

[18] Schneider HM, Lynch JP (2020). Should root plasticity be a crop breeding target?. Front. Plant Sci..

[19] Turner FT, Gilliam JW (1976). Increased P diffusion as an explanation of increased P availability in flooded rice soils. Plant Soil..

[20] Akahane I, Nanzyo M, Takahashi T, Sekiguchi O, Saigusa M (2006). Spatial distribution of phosphorus in the Ap horizon soils of paddy rice fields after rice harvest—Effect of machinery localized fertilizer application at transplanting of rice seedlings. Jpn. J. Soil Sci. Plant Nutr..

[21] Zhu J, Kaeppler SM, Lynch JP (2005). Topsoil foraging and phosphorus acquisition efficiency in maize (*Zea mays*). Funct. Plant Biol..

[22] Miguel MA, Postma JA, Lynch JP (2015). Phene synergism between root hair length and basal root growth angle for phosphorus acquisition. Plant Physiol..

[23] Sun B, Gao Y, Lynch JP (2018). Large crown root number improves topsoil foraging and phosphorus acquisition. Plant Physiol..

[24] Mori A (2016). The role of root size versus root efficiency in phosphorus acquisition of rice. J Exp. Bot..

[25] Arai-Sanoh Y (2014). Deep rooting conferred by DEEPER ROOTING 1 enhances rice yield in paddy fields. Sci. Rep..

[26] Rangarajan H, Postma JA, Lynch JP (2018). Co-optimization of axial root phenotypes for nitrogen and phosphorus acquisition in common bean. Ann. Bot..

[27] Sandhu N (2016). Rice root architectural plasticity traits and genetic regions of adaptability to variable cultivation and stress conditions. Plant Physiol..

[28] Rose TJ (2013). Enhancing phosphorus and zinc acquisition efficiency in rice: A critical review of root traits and their potential utility in rice breeding. Ann. Bot..

[29] Uga Y, Kitomi Y, Ishikawa S, Yano M (2015). Genetic improvement for root growth angle to enhance crop production. Breed. Sci..

[30] Oo AZ, Tsujimoto Y, Rakotoarisoa NM (2020). Optimizing the phosphorus concentration and duration of seedling dipping in soil slurry for accelerating the initial growth of transplanted rice. Agronomy.

[31] Motomizu S, Wakimoto T, Toei K (1983). Spectrophotometric determination of phosphate in river waters with molybdate and malachite green. Analyst..

[32] Murphy J, Riley JP (1962). A modified single solution method for the determination of phosphate in natural waters. Anal. Chem. Acta..

[33] Kano-Nakata M, Suralta RR, Niones JM, Yamauchi A, Shashidhar HE, Henry A, Hardy B (2012). Root sampling by using a root box-pinboard method. Methodologies for root drought studies in rice.

[34] Kano-Nakata M, Nakamura T, Mitsuya S, Yamauchi A (2019). Plasticity in root system architecture of rice genotypes exhibited under different soil water distributions in soil profile. Plant Prod Sci..

