# Supplementary material for: Synergy between a shallow root system with a DRO1 homologue and localized P application improves P uptake of lowland rice
Source: Sci Rep. 2021 May 4;11:9484. doi: 10.1038/s41598-021-89129-z (PMC8096825; doi:10.1038/s41598-021-89129-z)

**Synergy between a shallow root system with a *DRO1* homologue and localized P application improves P uptake of lowland rice**

Aung Zaw Oo^1#^, Yasuhiro Tsujimoto^1,#^ *, Mana Mukai^1^, Tomohiro Nishigaki^1^, Toshiyuki Takai^1^, Yusaku Uga^2^

^1^Japan International Research Center for Agricultural Sciences, 1-1 Ohwashi, Tsukuba, Ibaraki 3058686, Japan

^2^Institute of Crop Science, National Agriculture and Food Research Organization (NARO), 2-1-2, Kan-nondai, Tsukuba, Ibaraki 3058518, Japan

#These authors contributed equally to this work.

*Corresponding author. Tel/Fax: +81 29 838 6367; E-mail address: [tsjmt@affrc.go.jp](mailto:tsjmt@affrc.go.jp) (Yasuhiro Tsujimoto)

Supplementary information

Fig. S1 Horizontal and vertical root distribution patterns of three rice genotypes at 42 days after transplanting under the P-dipping (P_dip_) treatment. Root parameters were shown in the center and side positions of four different soil layers (0–3 cm, 3–7 cm, 7–14 cm, and 14–28 cm). The center position indicates 3 cm from the plant base in both horizontal directions. The side position is apart from the center position. Different small letters and capital letters indicate significant differences among genotypes in these parameters within each soil layer and in total of all layers, respectively, at 5% of Turkey`s HSD test. ns: not significant at 5% level.


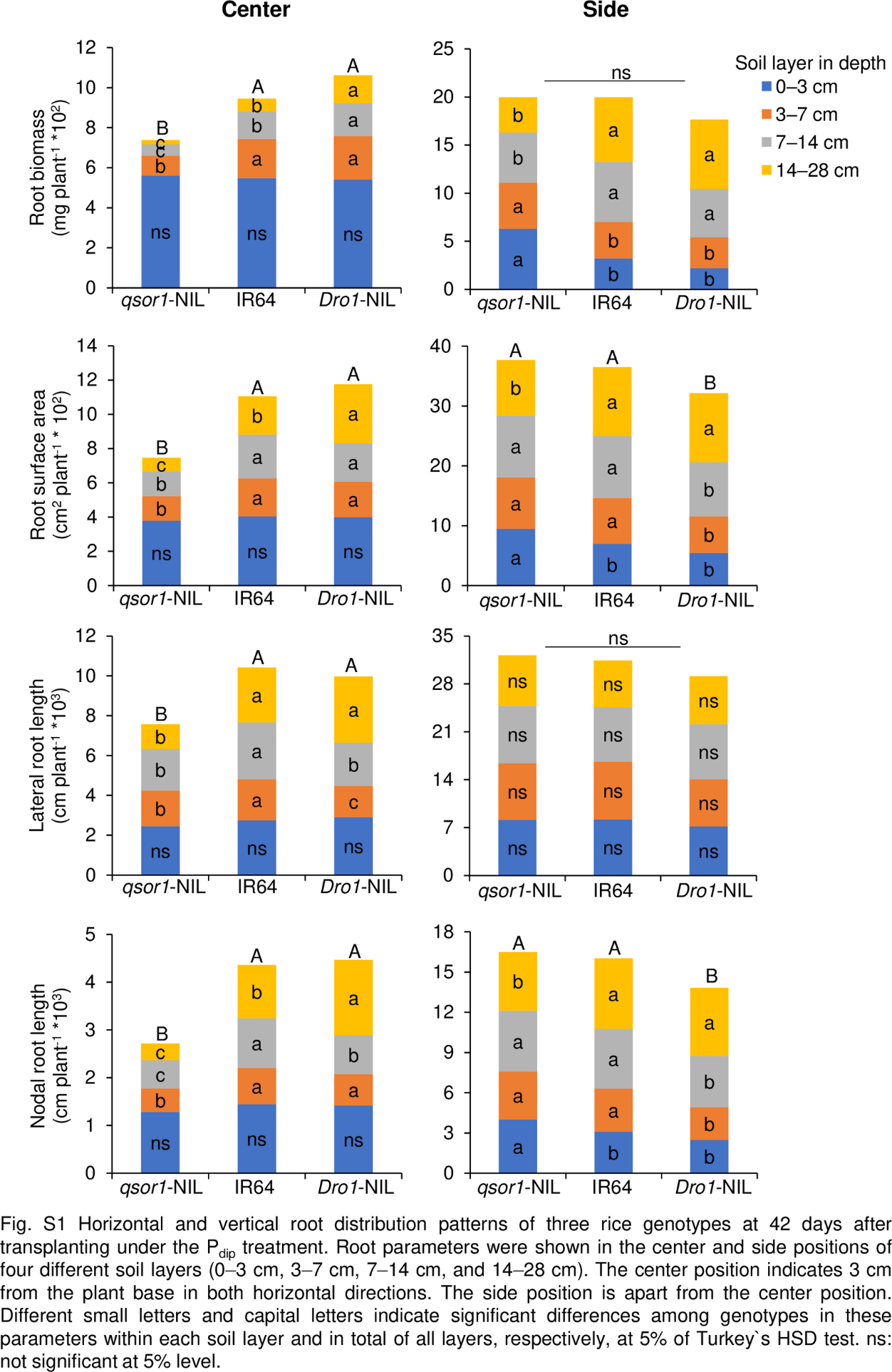

Supplement: Supplementary file 1 — Supplementary Information [file 41598_2021_89129_MOESM1_ESM.docx]
